# Mental health outcomes in pregnant women with relapsing-remitting multiple sclerosis: a longitudinal cohort study

**DOI:** 10.3389/fneur.2025.1619021

**Published:** 2025-07-15

**Authors:** Lena Kristina Pfeffer, Caren Ramien, Anja Harrison, Kostas Patas, Kristina Grentzenberg, Stefanie Reinhardt, Andrea Mönch, Max Kaufmann, Stefan M. Gold, Christoph Heesen

**Affiliations:** ^1^Institute of Neuroimmunology and Multiple Sclerosis, University Medical Center Hamburg-Eppendorf, Hamburg, Germany; ^2^Department of Neurology, University Medical Center Hamburg-Eppendorf, Hamburg, Germany; ^3^Department of Psychological Medicine, King’s College, London, United Kingdom; ^4^Department of Biopathology and Laboratory Medicine, Eginition Hospital, School of Medicine, National and Kapodistrian University of Athens, Athens, Greece; ^5^Department of Psychiatry and Neurosciences, Campus Benjamin Franklin, Charité – Universitätsmedizin Berlin, Berlin, Germany; ^6^Department of Psychosomatic Medicine, Campus Benjamin Franklin, Charité – Universitätsmedizin Berlin, Berlin, Germany

**Keywords:** multiple sclerosis, pregnancy, mental health, depression, stress, fatigue

## Abstract

**Introduction:**

Since multiple sclerosis (MS) primarily affects women of childbearing age, the disease intersects with a critical period for family planning and pregnancy. This is important, since pregnancy itself can influence psychological well-being, contributing to symptoms such as depression, stress and fatigue. However, while mental health during late pregnancy and the postpartum period has been studied in women with multiple sclerosis (wwMS), data on longitudinally tracking mental health in wwMS across all trimesters of pregnancy are still limited.

**Methods:**

In this prospective cohort study, we assessed the frequency and severity of depression, stress and fatigue in 95 women with relapsing-remitting MS (RRMS) throughout the course of pregnancy and postpartum using a set of psychological questionnaires. Furthermore, we evaluated the frequency and disease-specific risk factors of postpartum depression.

**Results:**

Over the course of pregnancy, there was no relevant increase in depressive symptoms, fatigue or stress. Moderate to high risk of postpartum depression was evident in 19.8% of wwMS and positively correlated with an increase in Expanded Disability Status Scale (EDSS) (*r* = 0.237, *p adj* = 0.049) during pregnancy but not with baseline EDSS.

**Discussion:**

Our data suggest that pregnancy does not generally increase the risk of stress, depression, or fatigue in wwMS, which is reassuring for both wwMS and their treating physicians. However, given the higher susceptibility to mental health alterations in MS, regular screening for mental health disturbances remains crucial. In particular, wwMS with disability progression during pregnancy should be closely monitored for postpartum depression.

## 1 Introduction

Multiple sclerosis (MS) is a chronic central nervous system (CNS) disorder affecting around 2.8 million individuals worldwide (1). It initially manifests as relapsing-remitting disease (RRMS) in 85–90% of cases (2). Typical symptoms include numbness, muscle weakness or visual problems; however, MS can also impact mental health, with anxiety, depression and fatigue being among the most common symptoms (3). Of note, these symptoms can be subtle and may therefore be overlooked. Since MS often begins between the ages of 20 and 40 years, its socioeconomic burden is substantial, and affected individuals often face significant uncertainties related to their life and family planning. Notably, women are affected by RRMS more than twice as often as men (4), making pregnancy planning and management a central issue in medical care. Historically, women with MS (wwMS) were often discouraged from having children due to concerns regarding disease and pregnancy outcomes. However, larger cohort studies revealed that relapse activity decreases during pregnancy (5). After delivery, relapse rate increases during the first 6 months, with the risk being reduced by pre-conceptional disease-modifying therapies (DMT) and exclusive breastfeeding (6). Managing DMT adjusted to the individual disease course is therefore crucial and facilitated by the increasing availability of DMT that are well manageable around, or even during pregnancy (7). Together, these insights have led to a paradigm shift regarding pregnancy management for wwMS, moving toward a personalized approach that encourages wwMS to realize their family planning, while carefully adjusting medical care based on their individual risk factors and needs. Maintaining stable mental health during pregnancy is essential for the well-being of both mother and child. Notably, the world health organization estimates that about 10% of pregnant women and 13% of women who have recently given birth will be affected by a mental health disorder (8). This is alarming since maternal mental health issues can increase the rate of pregnancy complications such as preterm labor (9). Furthermore, prenatal stress has been shown to modulate the brain and behavior of the offspring, although the underlying mechanisms are not yet fully understood (10). Studies investigating the effect of pregnancy on mental health have mainly focused on late pregnancy and the postpartum period (11, 12). Our study therefore aims to detailly examine the dynamics of depression, stress and fatigue across all trimesters of pregnancy until 3 months postpartum in order to gain deeper insights into the interaction between pregnancy and psychological well-being in the context of MS and to enable needs-adjusted care for wwMS at all stages of pregnancy.

## 2 Methods

### 2.1 Study design

All study participants were recruited prospectively from our MS outpatient clinic (Universitätsklinikum Hamburg-Eppendorf, Germany) between March 2011 and September 2024 based on the following inclusion criteria: (a) female individuals who were categorized as having RRMS who (b) either expressed a clear desire to have children in the near future or were in their first trimester of pregnancy. Retrospectively, all cases were re-evaluated for meeting the (13) criteria of RRMS (13) and all unclear cases were excluded to ensure accurate diagnosis.

Study inclusion was either performed before a planned pregnancy or during trimester 1. Depending on the time of inclusion in the study, there were 5 or 6 data collection points: (a) before pregnancy (pre), (b) trimester 1 during week 10–14 (tri 1), (c) trimester 2 during week 22–24 (tri 2), (d) trimester 3 during week 30–32 (tri 3), (e) two weeks postpartum (2 wpp), (f) 3 months postpartum (3 mpp). At inclusion, the following demographic and clinical information was collected by the medical team: year of birth, number of previous pregnancies, number of children, type of disease course, years from disease onset, years from diagnosis, Expanded Disability Status Scale (EDSS), previous and current DMT, concomitant medications and comorbidities. At all timepoints (pre, tri 1–3, 2 wpp, 3 mpp), psychological symptoms were assessed by a set of questionnaires [Perceived Stress Scale (PSS-14), Inventory of Depressive Symptomatology – self-reported (IDS-SR), Modified Fatigue Impact Scale (MFIS), Edinburgh Postnatal Depression Scale (EPDS)]. The questionnaires were distributed to the participants during their clinical visits, except 2 wpp, when they were sent to the participants as in-person visits were deemed unsuitable during the early postpartum period. At all clinical on-site visits (pre, tri 1–3, 3 mpp), clinical information regarding the occurrence and date of new relapses, current DMT and concomitant therapies as well as EDSS were assessed by the medical team. At 3 mpp, details regarding breastfeeding, sex of the child as well as postpartal medications were assessed via an additional questionnaire. Retrospectively, study participants with more than one missing timepoint between tri 1 and 3 mpp were excluded from the study. If individual questionnaires were missing or were too incomplete according to pre-defined criteria further described under *2.5*, the remaining completed questionnaires from that timepoint, as well as the corresponding questionnaires from other timepoints for that participant, were still included in the analysis.

The study was approved by the local ethics committee (Ethik-Kommission der Ärztekammer Hamburg, ethics committee vote PV3558) and informed written consent was given by all participants. Additionally, participants received a small compensation for time and effort after completing the last follow-up assessment. The study adhered to the Declaration of Helsinki.

### 2.2 Primary objective

The primary objective was to assess the severity and dynamics of depressive symptoms, fatigue and perceived stress in wwMS over the course of pregnancy and postpartum.

### 2.3 Secondary objective

The secondary objective was to evaluate disease-specific risk factors for depression, fatigue and stress during pregnancy as well as disease-specific risk factors for postpartum depression.

### 2.4 Questionnaires

Depressive symptoms were assessed using a validated German version (14) of the 30-item IDS-SR (15). In order to remove the impact of pregnancy and child care on weight and sleep and avoid potential misinterpretation, we adjusted the IDS total score for the present analysis: Originally, the score is calculated by adding the responses of 28 of the 30 items, ranging from 0 to 84 (none: 0–13, mild: 14–25, moderate: 26–38, severe: 39–48, very severe: ≥49). We removed question 2 (sleeping during the night), 4 (sleeping too much), as well as combined question 13 and 14 (gain or loss of weight). The modIDS used for the current analysis therefore contains 25 questions with the overall score ranging from 0 to 75. Cutoff values were adjusted accordingly (none: 0–12, mild: 13–22, moderate: 23–34, severe: 35–43, very severe: ≥44).

Postpartum depression was specifically assessed using the EPDS (16) in its validated German version (17). This questionnaire includes 10 questions with a Likert-type ranking from 0 to 3, with the overall score ranging from 0 to 30 (low probability of depression: 0–9, moderate probability of depression: 10–12, high probability of depression: ≥ 13).

The severity of fatigue symptoms was evaluated using the MFIS (18), which has been multinationally translated (19) and which is widely established in Germany (20) but not officially validated in its German translation. In this 21-item instrument, each item can be ranked on a scale from 0 to 4. Beside the total score (0–84), different sub-scores can be calculated to further specify between physical (0–36), cognitive (0–40) and psychosocial (0–8) aspects of fatigue. As a cutoff for the total score, one of the most commonly accepted values to discriminate between fatigued and non-fatigued individuals is 38 (19–21).

Furthermore, perceived stress was longitudinally assessed with a German translation of the 14-item PSS-14 (22), with its overall score ranging from 0 to 56. A validation of the German language version had only been conducted for the shortened 10-item version, confirming its reliability in a German-speaking population (23).

### 2.5 Statistical methods

Data analysis was performed using the *SPSS®* statistics software and *RStudio* (version 2023.6.1.524) with *R* version 4.2.2. Mean imputation was used in case of ≤10% missing values per questionnaire, by substituting missing items with the participant’s mean score from all completed items in that questionnaire. If missing values were above this cutoff, the respective questionnaires were excluded from the analysis. All statistical tests performed on the imputed datasets were similarly conducted on non-imputed control datasets only containing complete questionnaires, and are part of the Supplementary materials. For the assessment of disease-specific risk factors, linear regression analysis was performed. Changes in modIDS, MFIS and PSS-14 scores over the course of pregnancy and postpartum were analyzed using a linear mixed model (LMM) fitted with Restricted Maximum Likelihood (REML). For the main longitudinal analysis, the model included timepoints as fixed effects, with the intercept representing the baseline score at tri 1. Subsequent timepoints (tri 2, tri 3, 2 wpp, and 3 mpp) were included as factors to examine changes in relation to the tri 1 score. Additionally, a further longitudinal sub-group analysis was conducted, restricted to patients with available pre-pregnancy timepoints, using pre as the intercept to evaluate changes related to the pre timepoint. T tests with Satterthwaite’s approximation were used to account for the degrees of freedom. If the same hypothesis was tested on different variables (linear regression model, LMM), false discovery rate (FDR) adjustment of *p* values obtained from the analyses was conducted by Benjamini-Hochberg correction. Statistical tests performed on different questionnaires were considered as independent test families and were adjusted separately. The analyses were performed using the *lme4* and *lmerTest* packages in *Rstudio*. Statistical significance was defined as a *p* value less than 0.05, and this threshold was applied consistently across all analyses. For evaluating internal consistency of the PSS-14 and MFIS, Cronbach’s alpha was calculated using the *alpha ()* function from the *psych* package in *Rstudio*.

## 3 Results

### 3.1 Cohort characteristics

**Figure 1:**
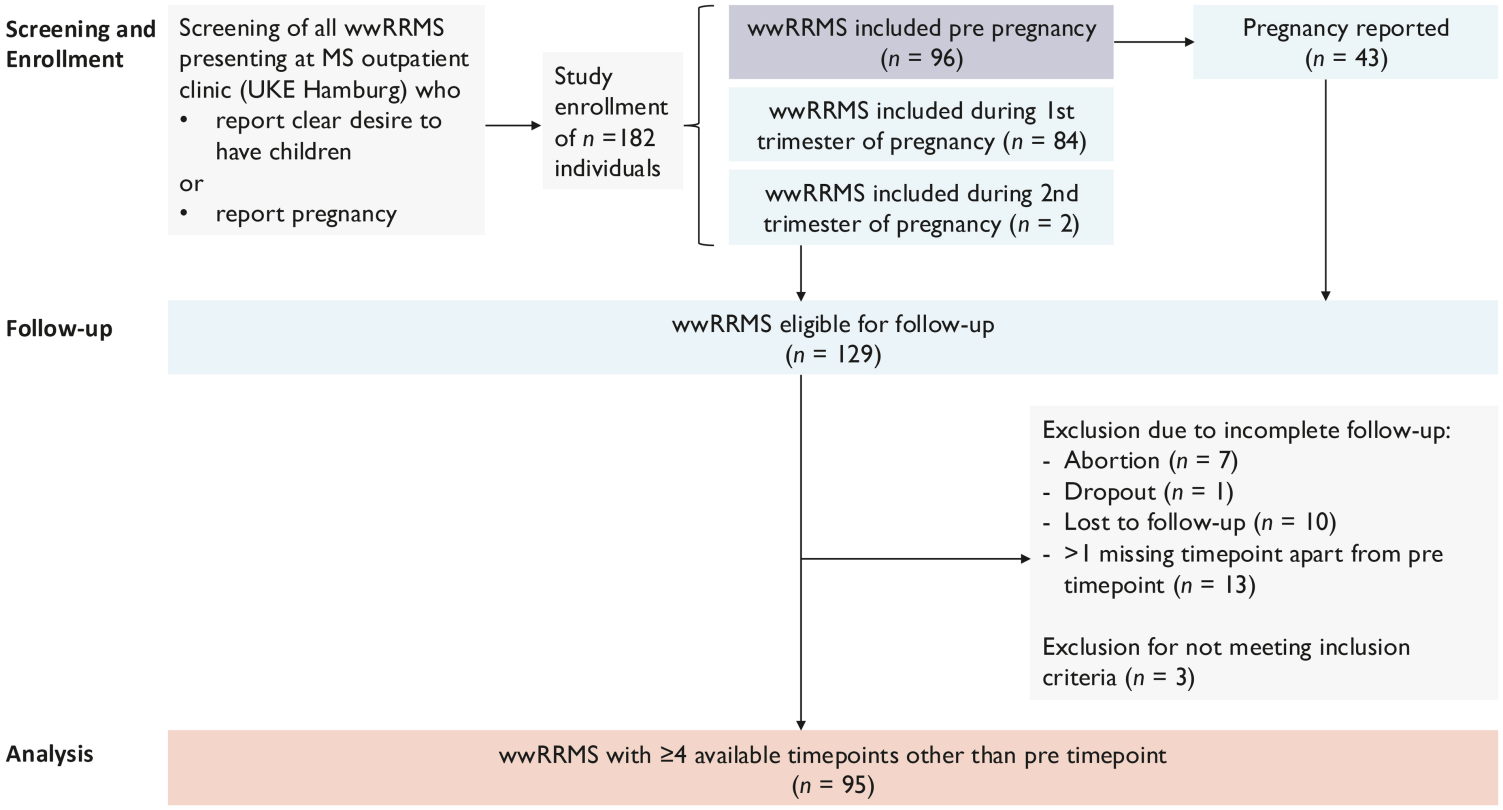
Recruitment strategy.

Between March 2011 and September 2024, a total of 182 wwMS were initially recruited for the study who either expressed a clear desire to have children in the near future (*n* = 96) or presented during tri 1 (*n* = 84). Two individuals were additionally included into the study at tri 2. Out of 182 individuals, 129 individuals reported a successful pregnancy, and these were subsequently followed up (Figure 1). During the follow-up period, 18 participants were lost to follow-up, attributed to miscarriage, withdrawal from the study, or lack of contactibility (Figure 1). Additionally, 16 participants were retrospectively excluded from the analysis as they either did not meet the (13) diagnostic criteria for RRMS (*n* = 3) or had more than one missing timepoint between tri 1 and 3 mpp (*n* = 13) (Figure 1).

**Figure 2:**
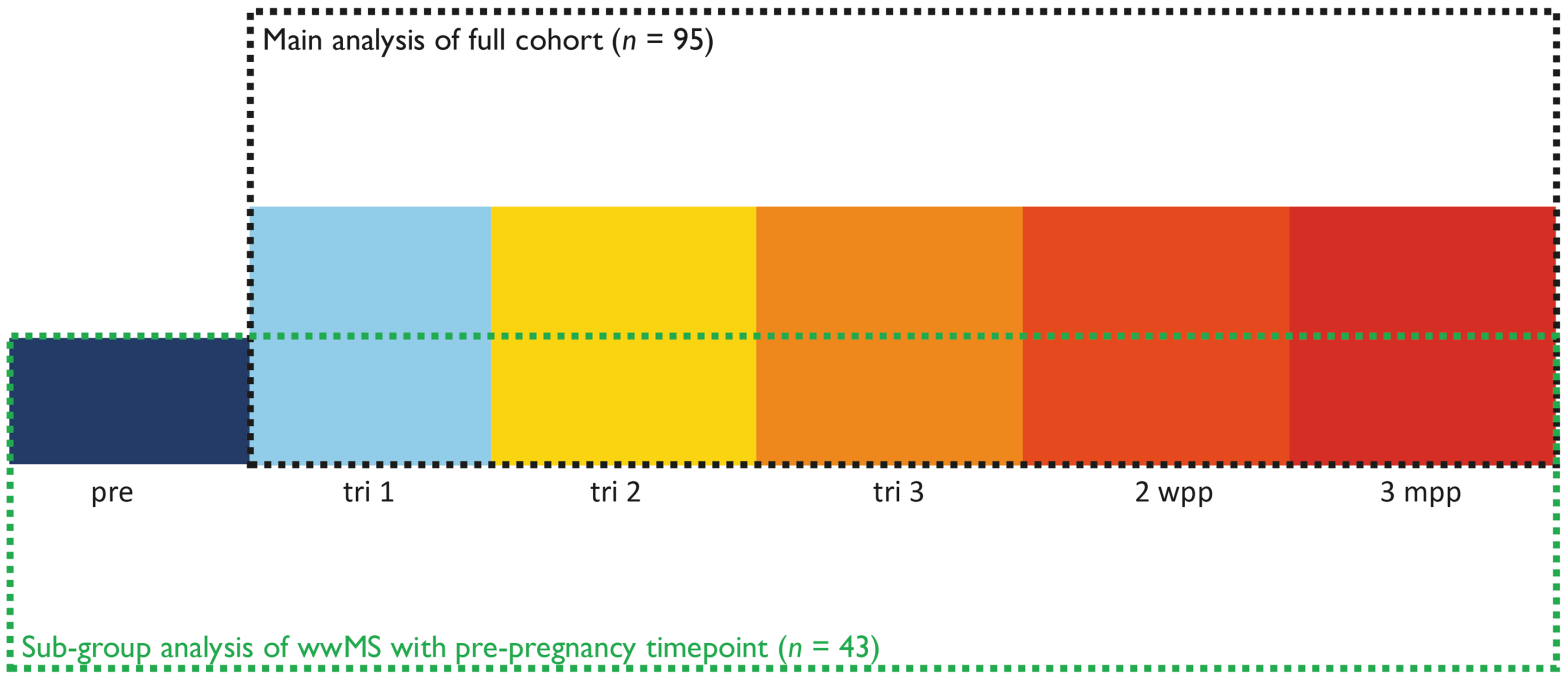
Overview of longitudinal analyses performed.

This resulted in a final cohort of 95 wwMS who were included in the analysis. Out of these study participants, 43 participants were enrolled before pregnancy and 50 participants during tri 1; two individuals were included during tri 2. Due to the limited number of participants included before pregnancy, the tri 1 timepoint was used as baseline timepoint for the analysis performed on all participants. However, a sub-group analysis of all participants with available pre timepoint was additionally performed. Figure 2 gives an overview about the two separate longitudinal analyses performed.

The mean age of the participants at the timepoint of tri 1 was 31.6 (SD = 4.5) years. For the majority of the participating women, it was their first pregnancy (63.2%). The median EDSS at tri 1 was 1.5 (range = 0.0–6.0), and 47.4% of the study participants had received DMT for MS within 6 months prior to pregnancy. Regarding disease progression over the course of pregnancy, 21.3% of wwMS showed neurological worsening (ΔEDSS ≥ + 0.5; min ΔEDSS = −2.0, max ΔEDSS = +4.0; ΔEDSS = EDSS at tri 3 – EDSS at tri 1) between tri 1 and 3, while 52.1% remained stable (ΔEDSS = 0) and 26.6% even had improvement (ΔEDSS ≤ − 0.5). Out of all participants showing disease progression, 63.0% had progression independent of relapses (PIRA) (24). The overall relapse rate over the whole course of pregnancy and postpartum was 29.5%. In detail, 12 participants (12.6%) experienced relapses during pregnancy, while 13 participants (13.7%) had relapses within the first 3 months postpartum. Additionally, three participants (3.2%) experienced relapses both during pregnancy and within the first 3 months postpartum. Further clinical and demographical characteristics are summarized in Table 1. Additionally, clinical and demographical characteristics of all wwMS with an available pre-pregnancy timepoint are also summarized in Table 1. Within this sub-group, the percentage of women experiencing their first pregnancy was higher (76.7%), while mean age (32.0 years, SD = 3.9 years) was comparable. Also, pre-pregnancy median EDSS (1.5, range = 0.0–4.5) and baseline median EDSS (1.5, range = 0.0–4.0) were representative for the main cohort. Neurological worsening between tri 1 and tri 3 occurred in 16.3% of participants. Mean duration between pre and tri 1 was 330.1 days (SD = 308.4).

**Table 1:** Pre-pregnancy, baseline and follow-up demographic and clinical characteristics.

Variable	Category/Value	Result	
		Full cohort (*n* = 95)	wwMS with pre-pregnancy timepoint (*n* = 43)
Age (tri 1)	Mean (SD)	31.6 (4.5)	32.0 (3.9)
	Range	20.0–40.0	23.0–40.0
Education level	Basic school^1^	9 (9.5%)	4 (9.3%)
	Secondary diploma^2^	18 (18.9%)	4 (9.3%)
	Technical diploma^3^	6 (6.3%)	2 (4.7%)
	High school diploma^4^	21 (22.1%)	11 (25.6%)
	University degree	41 (43.2%)	22 (51.2%)
Relationship status	In stable relationship	95 (100%)	43 (100%)
Number of previous pregnancies	0 (N/ %)	60 (63.2%)	33 (76.7%)
	1 (N/ %)	20 (21.1%)	5 (11.6%)
	2 (N/ %)	13 (13.7%)	4 (9.3%)
	3 (N/ %)	1 (1.1%)	1 (2.3%)
	6 (N/ %)	1 (1.1%)	–
Number of children	0 (N/ %)	69 (72.6%)	37 (86.0%)
	1 (N/ %)	20 (21.1%)	4 (9.3%)
	2 (N/ %)	5 (5.3%)	1 (2.3%)
	3 (N/ %)	1 (1.1%)	1 (2.3%)
Years from disease manifestation (tri 1)	Mean (SD)	5.4 (4.4)	5.3 (4.1)
	Range	0.0–18.0	0.0–18.0
Years from disease diagnosis (tri 1)	Mean (SD)	4.6 (4.1)	4.4 (3.9)
	Range	0.0–18.0	0.0–18.0
DMT < 6 months before pregnancy	Yes (N/ %)	45/ 47.4%	17/ 39.5%
	No (N/ %)	50/ 52.6%	26/ 60.5%
DMT during pregnancy	Yes (N/ %)	8/ 8.4%	3/ 7.0%
	No (N/ %)	87/ 91.6%	40/ 93.0%
DMT < 6 months after delivery	N-Miss (N/ %)	–	1/ 2.3%
	Yes (N/ %)	28/ 29.5%	10/ 23.3%
	No (N/ %)	67/ 70.5%	32/ 74.4
Concomitant medication (pre pregnancy)	Antidepressants (yes/no/NA in %)	3.2/ 55.8/ 41.1%	4.7/ 74.4/ 20.9%
	Anxiolytics (yes/no/NA in %)	1.1/ 55.8/ 43.2%	2.3/ 74.7/ 23.3%
	Painkillers (yes/no/NA in %)	2.1/ 55.8/ 42.1%	2.3/ 76.7/ 20.9%
Concomitant medication (tri 1 – tri 3)	Antidepressants (yes/no/NA in %)	0/ 63.2/ 36.8%	0/ 67.4/ 32.6%
	Anxiolytics (yes/no/NA in %)	0/ 63.2/ 36.8%	0/ 67.4/ 32.6%
	Painkillers (yes/no/NA in %)	0/ 63.2/ 36.8%	0/ 67.4/ 32.6%
Concomitant medication (within 3 mpp)	Antidepressants (yes/no/NA in %)	1.1/97.9/1.1%	2.3/ 95.3/ 2.3%
	Anxiolytics (yes/no/NA in %)	0/ 98.9/ 1.1%	0/ 97.7/ 2.3%
	Painkillers (yes/no/NA in %)	4.2/ 94.7/ 1.1%	2.3/ 95.3/ 2.3%
EDSS (pre)	N-Miss	–	2
	Median	NA	1.5
	Range	NA	0.0–4.5
Duration pre – tri 1	Mean (days)	–	330.1 (308.4)
EDSS (tri 1)	Median	1.5	1.5
	Range	0.0–6.0	0.0–4.0
EDSS (tri 3)	N-Miss	1	0
	Median	1.0	1.0
	Range	0.0–6.0	0.0–4.0
EDSS (3 mpp)	N-Miss	1	0
	Median	1.0	1.0
	Range	0.0–8.0	0.0–6.0
Occurence of relapses	Yes (N/ %)	28/ 29.5%	13/ 30.2%
	During pregnancy (N/ %)	12/ 12.6%	5/ 11.6%
	Postpartal (N/ %)	13/ 13.7%	6/ 14.0%
	During pregnancy + postpartal (N/%)	3/ 3.2%	2 / 4.7%
	No (N/ %)	67/ 70.5%	30/ 70.0%
Disease progression (tri 1 – tri 3)	N-Miss (N/ %)	1	0
	Increase of EDSS ≥ 0.5 (N/ % out of n = 94)	20/ 21.3%	7/ 16.3%
	Progression due to relapse	8/ 8.5%	3/ 7.0%
	Progression independent of relapses	12/ 12.8%	4/ 9.3%
	Stable EDSS (N/ %)	49/ 52.1%	26/ 60.5%
	Reduction of EDSS ≥ 0.5 (N/ %)	25/ 26.6%	10/ 23.3%

DMT = Disease-modifying therapy, EDSS = Expanded Disability Status Scale, N = number of participants, NA = not available, N-Miss = number of missing data points, SD = standard deviation, Tri = trimester, ^1^*Volks*- oder *Hauptschule* according to German school system, ^2^*Mittlere Reife* according to German school system, ^3^*Fachabitur* according to German school system, ^4^*Abitur* according to German school system.

### 3.2 Baseline assessment of depression, stress and fatigue during the first trimester

**Figure 3:**
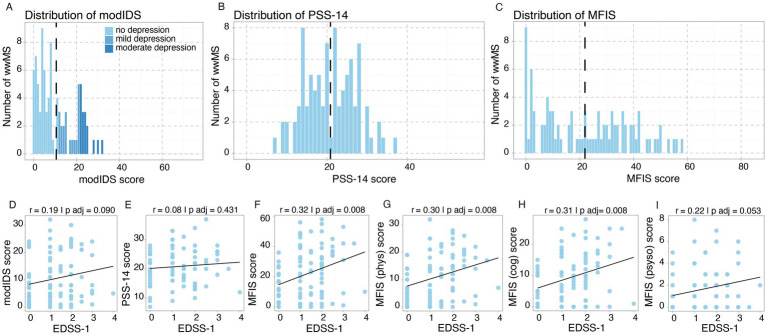
Baseline assessment of depression, stress and fatigue during tri 1. **(A–C)** Histograms illustrating frequency distribution of score values for **(A)** modIDS, **(B)** PSS-14 and **(C)** MFIS with mean values for each score depicted by intersected black vertical lines. **(D–I)** Correlation between baseline MS-related disability (EDSS-1) and **(D)** modIDS, **(E)** PSS-14, **(F)** MFIS and MFIS subscores [**(G)** physical, **(H)** cognitive, **(I)** psychosocial]. *p* values were adjusted for multiple comparisons using the *Benjamini-Hochberg* correction. Threshold for statistical significance: *p* < 0.05.

During tri 1, mean modIDS score was 10.6 (SD = 8.6), with 38.5% (no depression: *n* = 56/91, mild depression: *n* = 19/91, moderate depression: *n* = 16/91) of study participants experiencing mild or moderate depressive symptoms (Figure 3A; Table 2; Supplementary Table 1). Mean PSS-14 was 20.9 (SD = 6.4) at tri 1 (no cutoff available) (Figure 3B; Table 2; Supplementary Table 1) and mean MFIS was 22.0 (SD = 16.7, no cutoff applied) (Figure 3C; Table 2; Supplementary Table 1). While baseline depressive symptoms showed a slight trend toward a correlation with EDSS (*r* = 0.19, FDR-adjusted *p* value (*p adj*) = 0.090) (Figure 3D; Supplementary Figure 1A), there was no correlation between perceived stress (PSS-14) and EDSS (*r* = 0.08, *p adj* = 0.431) (Figure 3E; Supplementary Figure 1B). Baseline fatigue symptoms demonstrated the strongest positive correlation with baseline EDSS (*r* = 0.32, *p adj* = 0.008) (Figure 3F; Supplementary Figure 1C). In line with the other results, the correlation was stronger in the physical and cognitive domains of the MFIS compared to the psychosocial subscore (Figures 3G–I).

**Table 2:** Psychological assessment over time.

Score	Parameters	Tri 1	Tri 2	Tri 3	2 wpp	3 mpp
EPDS	questionnaires	88	93	93	91	90
	mean (SD)	5.0 (4.3)	4.6 (4.5)	4.7 (4.6)	5.5 (5.0)	4.4 (4.0)
	median	4.0	3.0	3.0	4.0	4.0
	range	0–17	0–18	0–19	0–22	0–15
modIDS	questionnaires	91	95	94	92	89
	mean (SD)	10.6 (8.6)	9.1 (7.3)	9.3 (7.8)	10.6 (11.0)	8.5 (8.5)
	median	8.0	8.0	8.0	7.0	7.0
	range	0–32	0–35	0–34	0–59	0–42
PSS-14	questionnaires	90	94	93	88	89
	mean (SD)	20.9 (6.4)	21.0 (6.5)	20.5 (7.1)	20.4 (7.5)	21.0 (6.7)
	median	20.5	21.0	20.0	21.0	21.0
	range	7–37	8–40	4–41	5–34	4–41
MFIS	questionnaires	91	93	93	92	90
	mean (SD)	22.0 (16.7)	20.9 (16.4)	22.3 (16.8)	23.6 (20.4)	20.6 (17.8)
	median	22.0	20.0	22.0	18.0	18.5
	range	0–58	0–58	0–61	0–71	0–63
MFIS (phys)	questionnaires	90	93	91	91	90
	mean (SD)	11.0 (8.4)	10.5 (7.8)	11.9 (8.4)	11.9 (10.1)	9.0 (8.4)
	median	10.5	11.0	13.0	10.0	7.0
	range	0–32	0–27	0–34	0–36	0–28
MFIS (cog)	questionnaires	91	94	93	88	90
	mean (SD)	9.3 (7.9)	9.2 (8.2)	9.0 (8.2)	9.5 (9.6)	10.2 (8.7)
	median	9.0	8.0	8.0	6.0	9.5
	range	0–28	0–31	0–26	0–32	0–32
MFIS (psyso)	questionnaires	92	93	93	92	90
	mean (SD)	1.6 (1.9)	1.3 (1.4)	1.4 (1.8)	2.1 (2.3)	1.5 (1.9)
	median	1.0	1.0	0.0	1.5	0.5
	range	0–8	0–5	0–8	0–8	0–6

2 wpp = 2 weeks postpartum, 3 mpp = 3 months postpartum, EPDS = Edinburgh Postnatal Depression Scale (EPDS), MFIS = Modified Fatigue Impact Scale (cog = cognitive, phys = physical, psyso = psychosocial), modIDS = modified Inventory for Depressive Symptomatology, PSS-14 = Perceived Stress Scale.

### 3.3 Dynamics of depression, stress and fatigue over the course of pregnancy and postpartum

**Figure 4:**
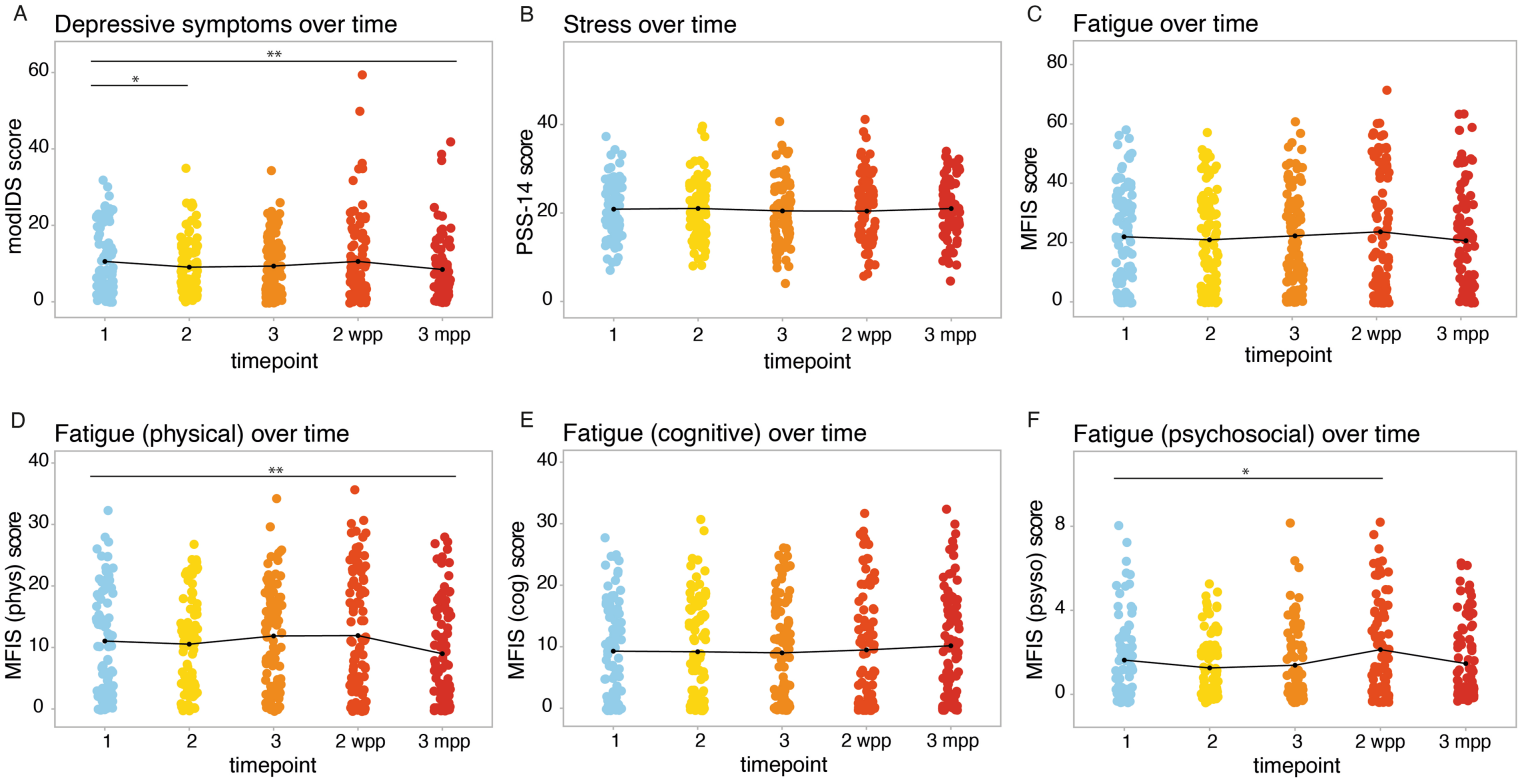
Dynamics of depression, stress and fatigue over the course of pregnancy. Psychometric scores were assessed at tri 1 (1), tri 2 (2), tri 3 (3), 2 wpp and 3 mpp, with mean values per timepoint indicated by black curves: **(A)** modIDS, **(B)** PSS-14, **(C)** MFIS, MFIS **(D)** physical, **(E)** cognitive, **(F)** psychosocial subscores. Significance levels: *p < 0.05, **p < 0.01.

Over the course of pregnancy and postpartum, a slight decrease in depression compared to baseline (tri 1) was observed. This decrease was statistically significant (threshold: *p* < 0.05) between tri 1 and tri 2 (*p adj* = 0.04) and between tri 1 and 3 mpp (*p adj* = 0.003), while at 2 wpp there was no statistically significant change (Figure 4A; Tables 2, 3; Supplementary Tables 1, 2). Stress symptoms remained stable over the course of pregnancy and during the postpartum period, with no statistically significant changes observed (Figure 4B; Tables 2, 3; Supplementary Tables 1, 2). The level of fatigue remained largely stable compared to baseline and only showed slight, statistically non-significant fluctuations (Figure 4C; Tables 2, 3; Supplementary Tables 1, 2). When looking at fatigue sub-scores, a decline in physical fatigue at 3 mpp compared to baseline was notable, while psychosocial fatigue showed a statistically significant increase within the first 2 wpp (*p* = 0.023). Cognitive fatigue, on the other hand, remained stable (Figures 4D–F; Tables 2, 3; Supplementary Tables 1, 2). Since the PSS-14 and MFIS are not validated in German language, Cronbach’s alpha was calculated separately at all timepoints from tri 1–3 mpp as indicator of validity and ranged between 0.67–0.76 (PSS-14) and between 0.96–0.97 (MFIS).

**Table 3:** Analysis of psychometric score dynamics with LMM.

Timepoint	Estimate	Std. Error	df	t value	Pr(>|t|) adj	Significance level
modIDS						
Intercept (Tri 1)	10.74	0.91	173.12	11.85	(< 2^−16^)	(***)
Timepoint 2 (Tri 2)	−1.68	0.74	362.38	−2.27	0.040	*
Timepoint 3 (Tri 3)	−1.44	0.75	362.56	−1.93	0.068	ns
Timepoint 4 (2 wpp)	−0.25	0.75	362.71	−0.33	0.744	ns
Timepoint 5 (3 mpp)	−2.50	0.76	363.13	−3.29	0.003	**
PSS-14						
Intercept (Tri 1)	20.72	0.71	204.67	29.17	(<2^−16^)	(***)
Timepoint 2 (Tri 2)	0.31	0.65	357.23	0.48	0.745	ns
Timepoint 3 (Tri 3)	−0.27	0.66	357.48	−0.41	0.745	ns
Timepoint 4 (2 wpp)	−0.22	0.67	357.39	−0.33	0.745	ns
Timepoint 5 (3 mpp)	0.26	0.67	358.12	0.39	0.745	ns
MFIS						
Intercept (Tri 1)	21.85	1.82	144.62	12.02	(<2^−16^)	(***)
Timepoint 2 (Tri 2)	−0.98	1.28	360.57	−0.77	0.555	ns
Timepoint 3 (Tri 3)	0.12	1.28	360.67	0.10	0.925	ns
Timepoint 4 (2 wpp)	1.70	1.28	360.64	1.33	0.307	ns
Timepoint 5 (3 mpp)	−1.93	1.29	360.89	−1.50	0.307	ns
MFIS (phys)						
Intercept (Tri 1)	11.05	0.89	163.04	12.38	(< 2^−16^)	(***)
Timepoint 2 (Tri 2)	−0.53	0.70	358.91	−0.76	0.446	ns
Timepoint 3 (Tri 3)	0.69	0.70	359.05	0.98	0.409	ns
Timepoint 4 (2 wpp)	0.86	0.70	358.86	1.22	0.372	ns
Timepoint 5 (3 mpp)	−2.38	0.71	359.35	−3.37	0.003	**
MFIS (cog)						
Intercept (Tri 1)	9.23	0.88	143.55	10.50	(<2^−16^)	(***)
Timepoint 2 (Tri 2)	−0.10	0.61	357.49	−0.16	0.871	ns
Timepoint 3 (Tri 3)	−0.37	0.61	357.66	−0.60	0.683	ns
Timepoint 4 (2 wpp)	0.45	0.62	357.93	0.72	0.683	ns
Timepoint 5 (3 mpp)	0.58	0.62	357.88	0.94	0.683	ns
MFIS (psyso)						
Intercept (Tri 1)	1.61	0.19	232.47	8.36	(5.62^−15^)	(***)
Timepoint 2 (Tri 2)	−0.34	0.19	362.48	−1.79	0.123	ns
Timepoint 3 (Tri 3)	−0.24	0.19	362.70	−1.230	0.275	ns
Timepoint 4 (2 wpp)	0.51	0.19	362.67	2.634	0.023	*
Timepoint 5 (3 mpp)	−0.17	0.19	363.26	−0.879	0.380	ns

df = degrees of freedom, Pr(>|t|) adj = p value corresponding to t-statistic for each fixed effect (FDR adjustment with Benjamini-Hochberg correction), Std. error = standard error. Significance levels: * = *p* < 0.05, ** = *p* < 0.01, *** = *p* < 0.001, ns = not significant.

A sub-group analysis of questionnaires of all wwMS with an available pre-pregnancy timepoint (*n* = 43) revealed a statistically not significant reduction of depressive symptoms, stress symptoms as well as of the overall MFIS score between pre pregnancy and tri 1 (Tables 4, 5; Supplementary Tables 3, 4; Supplementary Figure 2). Analyzing the dynamics of modIDS over the course of pregnancy, there was a significant reduction between pre and tri 2 (*p adj* = 0.010), pre and tri 3 (*p adj* = 0.048) as well as pre and 3 mpp (*p adj* = 0.006) (Table 5; Supplementary Tables 3, 4). The level of stress and fatigue remained stable, with no statistically significant changes over the course of pregnancy and postpartum (Table 5).

**Table 4:** Psychological assessment over time – sub-group analysis of wwMS with available pre-pregnancy timepoint.

Score	Parameters	Pre	Tri 1	Tri 2	Tri 3	2 wpp	3 mpp
EPDS	questionnaires	41	40	40	40	40	38
	mean (SD)	5.8 (5.4)	4.9 (4.4)	3.7 (3.6)	4.5 (4.0)	5.5 (4.7)	4.3 (3.6)
	median	5.0	4.0	3.0	3.0	4.5	4.0
	range	0–24	0–17	0–12	0–13	0–18	0–15
modIDS	questionnaires	42	41	42	41	42	40
	mean (SD)	11.6 (9.3)	10.4 (8.6)	8.5 (5.8)	9.3 (6.7)	10.2 (10.3)	8.4 (7.3)
	median	0–38	0–30	0–20	0–24	0–50	0–39
	range	10.5	7.0	8.0	8.0	7.0	8.0
PSS-14	questionnaires	41	40	41	40	40	39
	mean (SD)	21.6 (5.7)	20.5 (6.3)	20.8 (6.3)	20.3 (6.6)	20.9 (7.6)	20.1 (6.7)
	median	22.0	21.5	21.0	20.5	22.0	20.0
	range	10–32	9–34	11–40	8–34	6–41	8–34
MFIS	questionnaires	40	39	39	39	40	38
	mean (SD)	24.1 (20.3)	23.6 (17.5)	21.8 (14.7)	23.6 (16.9)	23.7 (21.2)	22.0 (18.6)
	median	16.0	22.0	25.0	27.0	20.5	17.5
	range	0–62	0–58	0–57	0–61	0–71	0–63
MFIS (phys)	questionnaires	40	39	39	39	40	38
	mean (SD)	10.9 (9.8)	11.7 (8.3)	10.6 (7.1)	11.6 (8.4)	11.2 (10.0)	9.0 (8.5)
	median	7.5	12.0	12.0	15.0	9.5	6.5
	range	0–29	0–28	0–24	0–30	0–31	0–28
MFIS (cog)	questionnaires	40	39	40	39	38	38
	mean (SD)	11.5 (9.4)	10.1 (8.6)	9.9 (7.6)	10.5 (8.3)	10.5 (10.2)	11.8 (9.2)
	median	12.0	10.0	11.5	10.0	9.5	11.0
	range	0–30	0–28	0–31	0–25	0–32	0–30
MFIS (psyso)	questionnaires	40	39	40	39	40	38
	mean (SD)	1.7 (1.9)	1.8 (2.0)	1.4 (1.4)	1.5 (1.9)	2.0 (2.3)	1.2 (1.7)
	median	1.0	2.0	1.0	1.0	1.0	0.0
	range	0–5	0–7	0–5	0–8	0–8	0–5

2 wpp = 2 weeks postpartum, 3 mpp = 3 months postpartum, EPDS = Edinburgh Postnatal Depression Scale (EPDS), MFIS = Modified Fatigue Impact Scale (cog = cognitive, phys = physical, psyso = psychosocial), modIDS = modified Inventory for Depressive Symptomatology, PSS-14 = Perceived Stress Scale.

**Table 5:** Analysis of psychometric score dynamics with LMM – sub-group analysis of wwMS with available pre-pregnancy timepoint.

Timepoint	Estimate	Std. Error	df	t value	Pr(>|t|) adj	Significance level
modIDS						
Intercept (Pre)	11.62	1.26	85.23	9.25	(1.66^−14^)	(***)
Timepoint 2 (Tri 1)	−1.27	1.11	201.16	−1.14	0.255	ns
Timepoint 3 (Tri 2)	−3.12	1.11	201.06	−2.82	0.010	*
Timepoint 4 (Tri 3)	−2.41	1.11	201.21	−2.16	0.048	*
Timepoint 5 (2 wpp)	−1.43	1.11	201.06	−1.29	0.238	ns
Timepoint 6 (3 mpp)	−3.51	1.12	201.32	−3.13	0.006	**
PSS-14						
Intercept (Pre)	21.63	1.02	99.44	21.25	(<2^−16^)	(***)
Timepoint 2 (Tri 1)	−1.14	0.99	195.47	−1.15	0.378	ns
Timepoint 3 (Tri 2)	−0.88	0.99	195.26	−0.89	0.441	ns
Timepoint 4 (Tri 3)	−1.42	0.99	195.47	−1.43	0.308	ns
Timepoint 5 (2 wpp)	−0.77	0.99	195.47	−0.77	0.441	ns
Timepoint 6 (3 mpp)	−1.64	1.00	195.61	−1.63	0.308	ns
MFIS						
Intercept (Pre)	24.07	2.90	60.28	8.30	(1.48^−11^)	(***)
Timepoint 2 (Tri 1)	−0.78	2.03	190.06	−0.39	0.834	ns
Timepoint 3 (Tri 2)	−2.30	2.04	190.06	−1.13	0.522	ns
Timepoint 4 (Tri 3)	−0.85	2.04	190.09	−0.42	0.834	ns
Timepoint 5 (2 wpp)	−0.42	2.02	190.01	−0.21	0.834	ns
Timepoint 6 (3 mpp)	−3.10	2.05	190.15	−1.51	0.396	ns
MFIS (phys)						
Intercept (Pre)	10.87	1.38	66.21	7.87	(2.66^−10^)	(***)
Timepoint 2 (Tri 1)	0.66	1.06	190.09	0.62	0.809	ns
Timepoint 3 (Tri 2)	−0.26	1.06	190.09	−0.24	0.809	ns
Timepoint 4 (Tri 3)	0.59	1.06	190.12	0.56	0.809	ns
Timepoint 5 (2 wpp)	0.35	1.05	190.01	0.33	0.809	ns
Timepoint 6 (3 mpp)	−2.35	1.07	190.19	−2.20	0.087	ns
MFIS (cog)						
Intercept (Pre)	11.52	1.41	62.79	8.16	(1.16^−10^)	(***)
Timepoint 2 (Tri 1)	−1.55	1.03	189.04	−1.50	0.272	ns
Timepoint 3 (Tri 2)	−1.62	1.03	188.98	−1.58	0.272	ns
Timepoint 4 (Tri 3)	−1.26	1.03	189.07	−1.22	0.336	ns
Timepoint 5 (2 wpp)	−0.93	1.04	189.11	−0.89	0.451	ns
Timepoint 6 (3 mpp)	−0.20	1.04	189.14	−0.19	0.852	ns
MFIS (psyso)						
Intercept (Pre)	1.68	0.30	99.36	5.63	(9.0.89^−7^)	(***)
Timepoint 2 (Tri 1)	0.11	0.29	191.23	0.38	0.702	ns
Timepoint 3 (Tri 2)	−0.33	0.29	191.08	−1.11	0.521	ns
Timepoint 4 (Tri 3)	−0.18	0.29	191.29	−0.60	0.659	ns
Timepoint 5 (2 wpp)	0.27	0.29	191.08	0.94	0.521	ns
Timepoint 6 (3 mpp)	−0.54	0.30	191.44	−1.83	0.207	ns

df = degrees of freedom, Pr(>|t|) adj = p value corresponding to t-statistic for each fixed effect (FDR adjustment with Benjamini-Hochberg correction), Std. error = standard error. Significance levels: * = *p* < 0.05, ** = *p* < 0.01, *** = *p* < 0.001, ns = not significant.

### 3.4 Assessment and disease-specific risk factors of postpartum depressive symptoms

**Figure 5:**
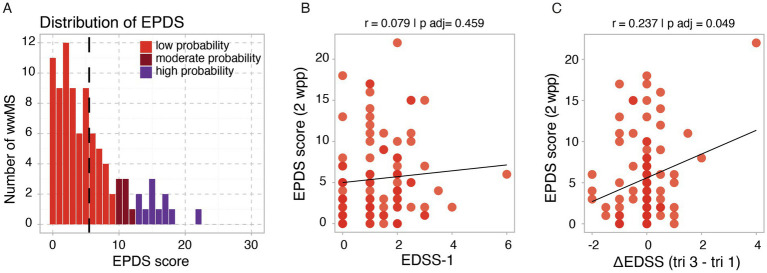
Assessment and disease-specific correlates of postpartum depression. **(A)** Histograms illustrating frequency distribution of EPDS score values at 2 wpp. Correlation between **(B)** baseline EDSS at tri 1 and **(C)** ΔEDSS (tri 3 - tri 1) with EPDS at 2 wpp. Threshold for statistical significance: *p* < 0.05.

Postpartum depressive symptoms were assessed 2 wpp using the EPDS score. On average, participants had a mean EPDS score of 5.5 (SD = 5.0) (Figure 5A), with 19.8% of participants showing moderate to high probability of postpartum depression (low probability of depression: *n* = 73/91, moderate probability of depression: *n* = 7/91, high probability of depression: n = 11/91). While there was no correlation of EPDS score with baseline EDSS at tri 1 (*r* = 0.079, *p adj* = 0.459) (Figure 5B), a positive correlation with disability worsening over the course of pregnancy (ΔEDSS) was notable (*r* = 0.237, *p adj* = 0.049) (Figure 5C).

## 4 Discussion

In this study, we investigated the frequency and dynamics of depressive symptoms (modIDS, EPDS), perceived stress (PSS-14) and fatigue (MFIS) in wwMS during pregnancy and the postpartum period. Within a smaller sub-group analysis of all wwMS that started the longitudinal follow-up pre pregnancy (*n* = 43), we also analyzed changes of these scores starting from the pre-pregnancy timepoint. Additionally, we analyzed whether MS-related disability and disease progression during pregnancy impact these psychological symptom domains.

In the first trimester, 38.5% of wwMS experienced mild to severe depressive symptoms according to the modIDS, with a mean modIDS score of 10.6 (SD = 8.6) across all study participants. Data from the general population based on data of *n* = 1,295 subjects with the 30-item IDS-SR version reveal a mean score of 6.7 (SD = 6.9) (25), suggesting that our study participants display an increased level of depressive symptoms compared to the general population. Perinatal depressive symptoms can be aggravated by both pregnancy and MS: The prevalence of depression during the second and third trimester of healthy pregnant women has been reported to be twice as high as in the general female population (26). In MS, depression is known to be increased, with a lifetime prevalence of 40 to 60% which is 3 to 10 times higher than that of the general population (27, 28). This matches with the finding that during the third trimester, wwMS were shown to be more frequently affected by depression than heathy pregnant women (15% vs. 9%) (11). It remains of interest whether pregnancy-specific mechanisms contribute to the increased risk of antenatal depression in MS or if these numbers solely reflect the MS-related risk. Our study indicates that depressive symptoms tend to remain relatively stable throughout pregnancy. Our sub-group analysis of 43 wwMS with available pre-pregnancy timepoint even displayed a slight reduction of depressive symptoms from pre pregnancy until tri 1, supporting the latter hypothesis. However, studies including larger pre-pregnancy data will be needed to fully assess this question.

Postpartum depression was specifically assessed using the EPDS score. At 2 wpp, 19.8% of wwMS showed signs of moderate to high probability of postpartum depression, while 12.1% demonstrated a high probability of postpartum depression. This result aligns with a retrospective study by Krysko et al., which identified postpartum depression in 18 out of 143 pregnancies (12.6%) among wwMS (12) and is comparable with the healthy population (29). At 3 mpp, mean EPDS already decreased compared to the 2 wpp score. Notably, the risk of postpartum depression did not correlate with baseline EDSS but positively correlated with worsening disability over the course of pregnancy, indicating that insufficient disease control might also increase the risk of postpartum depression. In principle, it would also be of interest to separately analyze how relapses during pregnancy and the early postpartum period influence the risk of postpartum depression, as an acute deterioration might be perceived as particularly destabilizing. However, the overall incidence of relapses in our cohort was too low, and their occurrence was too temporally dispersed, which is why this analysis was not included in the manuscript. In the future, the psychological consequences of relapses during pregnancy should be evaluated in larger cohorts. Another disease-specific risk factor for postpartum depression would be the impact of an early initiation of immunotherapy after pregnancy. Additionally, in terms of potential prevention, it would be relevant to investigate the influence of lifestyle factors such as physical activity and relaxation exercises.

Despite expectations of fluctuating mental health during pregnancy and postpartum, stress and total fatigue scores remained stable throughout. Regarding stress, the maximum PSS-14 score throughout the entire pregnancy was 21.0 (SD = 6.5), which is comparable to age-matched US-American cohorts from the general population (30). Direct comparisons of the dynamics of stress symptoms during pregnancy between wwMS and healthy pregnant women have not been performed. However, there is data from a healthy Arabic cohort that found no significant differences of the Arabic version of the PSS-10 between pregnant and postpartum women (31). Regarding fatigue symptoms, study participants scored lower than other MS cohorts (21). A positive correlation between MS-related disability and fatigue symptoms has been reported (32, 33), which we also found in our cohort. Therefore, the lower levels of fatigue in our cohort could be explained by the relatively high proportion of study participants with only mild or no disability. However, it is nevertheless remarkable that pregnancy-related physiological changes did not lead to an increased fatigue in wwMS over the course of pregnancy.

This study has some important limitations to be considered. The small sample size limited our ability to perform more stratified analyses, such as evaluating the impact of DMT or relapses as risk factors for postpartum depression. Additionally, due to limited pre-pregnancy data, the baseline timepoint needed to be set at tri 1 for the main analysis, which does not fully capture the baseline situation before pregnancy. As another limitation, stress and fatigue were assessed using questionnaires that have only partly been validated in German language. Furthermore, no matched healthy controls were studied. Lastly, it should be noted that the cohort might not be fully representative, as overall neurological impairment was low, and the participants had a relatively high level of education compared to the general German population (34).

## 5 Conclusion

In summary, our data indicate that mental health remains mostly stable during pregnancy in wwMS, which is encouraging both for wwMS and treating physicians dealing with the topic of pregnancy. Our data also illustrate once again that MS is an important risk factor for depression, which should not be neglected even during pregnancy, as it can represent an additional potential trigger. Moreover, special precautions are essential for wwMS with persisting disease activity during pregnancy.

## Data Availability

The raw data supporting the conclusions of this article will be made available by the authors, without undue reservation.
