# Supplementary material for: Mental health outcomes in pregnant women with relapsing-remitting multiple sclerosis: a longitudinal cohort study
Source: Front Neurol. 2025 Jul 15;16:1619021. doi: 10.3389/fneur.2025.1619021 (PMC12305591; doi:10.3389/fneur.2025.1619021)
Supplement: Supplementary file 1 [file Data_Sheet_1.pdf]

Supplementary material

Mental health outcomes in pregnant women with relapsing-remitting multiple sclerosis: A longitudinal cohort study

Authors

Lena Kristina Pfeffer, Caren Ramien, Anja Harrison, Kostas Patas, Kristina Grentzenberg, Stefanie Reinhardt, Andrea Mönch, Max Kaufmann, Stefan M. Gold, Christoph Heesen

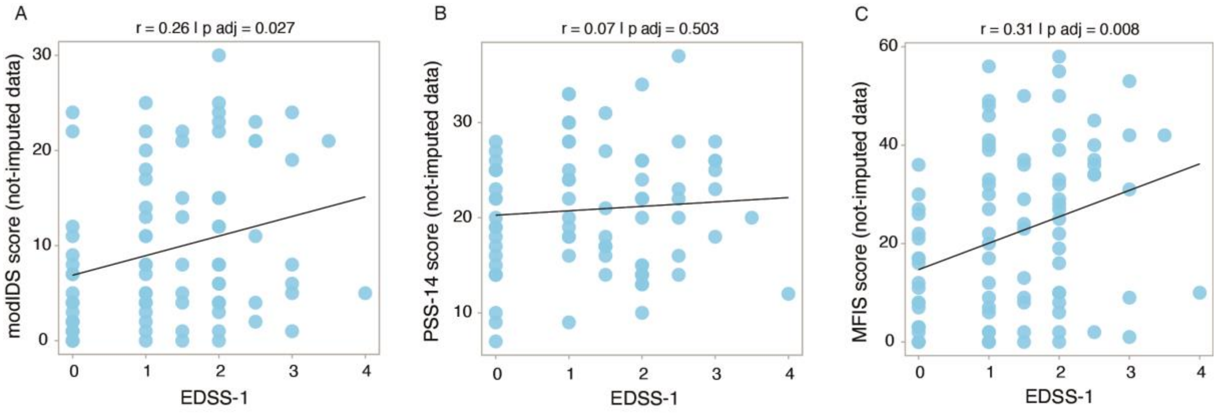

Suppl. Figure 1. Correlation analysis (not-imputed data). Correlation between (A) modIDS, (B) PSS-14 and (C) MFIS and EDSS-1 (EDSS at tri 1). *p* values were adjusted for multiple comparisons using the *Benjamini-Hochberg* correction. Threshold for statistical significance: $p < 0.05$.

Suppl. Table 1. Psychological assessment over time (not-imputed data)

Score	Parameters	Tri 1	Tri 2	Tri 3	2 wpp	3 mpp
modIDS	questionnaires	84	91	90	85	82
	mean (SD)	9.7 (8.1)	8.5 (6.7)	8.9 (7.3)	10.3 (11.0)	8.4 (8.7)
	median	7.5	7.0	7.5	7.0	6.0
	range	0-30	0-26	0-26	0-59	0-42
PSS-14	questionnaires	84	89	87	87	87
	mean (SD)	20.9 (6.4)	21.0 (6.5)	20.3 (6.9)	20.5 (7.5)	20.9 (6.6)
	median	20.5	21.0	20.0	21.0	21.0
	range	7-37	8-40	4-41	6-41	5-33
MFIS	questionnaires	90	92	93	87	90
	mean (SD)	21.8 (16.8)	20.9 (16.5)	22.3 (16.8)	23.3 (20.6)	20.6 (17.8)
	median	21.5	20.0	22.0	17.0	18.5
	range	0-58	0-57	0-61	0-71	0-63

Abbreviations: 2 wpp = two weeks postpartum, 3 mpp = three months postpartum, EPDS = Edinburgh Postnatal Depression Scale (EPDS), MFIS = Modified Fatigue Impact Scale (cog = cognitive, phys = physical, psyso = psychosocial), modIDS = modified Inventory for Depressive Symptomatology, PSS-14 = Perceived Stress Scale

Suppl. Table 2. Analysis of psychometric score dynamics with LMM (not-imputed data)

Timepoint	Estimate	Std. Error	df	t value	Pr(>|t|) adj	Significance level
**modIDS**						
**Intercept (Tri 1)**	10.45	0.91	165.94	11.45	(< 2^{-16})	(***)
**Timepoint 2 (Tri 2)**	-1.65	0.73	333.46	-2.26	0.042	*
**Timepoint 3 (Tri 3)**	-1.41	0.73	333.79	-1.92	0.069	ns
**Timepoint 4 (2 wpp)**	-0.46	0.75	334.41	-0.62	0.537	ns
**Timepoint 5 (3 mpp)**	-2.33	0.76	334.98	-3.08	0.005	**
**PSS-14**						
**Intercept (Tri 1)**	20.68	0.72	215.27	28.65	(<2^{-16})	(***)
**Timepoint 2 (Tri 2)**	0.34	0.68	338.81	0.50	0.863	ns
**Timepoint 3 (Tri 3)**	-0.23	0.69	339.05	-0.33	0.863	ns
**Timepoint 4 (2 wpp)**	-0.12	0.69	338.66	-0.17	0.863	ns
**Timepoint 5 (3 mpp)**	0.24	0.69	340.18	0.35	0.863	ns
**MFIS**						
**Intercept (Tri 1)**	21.90	1.82	145.46	12.02	(<2^{-16})	(***)
**Timepoint 2 (Tri 2)**	-1.06	1.29	353.90	-0.83	0.510	ns
**Timepoint 3 (Tri 3)**	0.07	1.28	353.93	0.06	0.955	ns
**Timepoint 4 (2 wpp)**	1.85	1.31	353.89	1.42	0.263	ns
**Timepoint 5 (3 mpp)**	-1.98	1.30	354.16	-1.53	0.263	ns

Abbreviations: df = degrees of freedom, Pr(>|t|) adj = *p* value corresponding to t-statistic for each fixed effect (FDR adjustment with Benjamini-Hochberg correction), Std. error = standard error. Significance levels: * = $p < 0.05$, ** = $p < 0.01$, *** = $p < 0.001$, ns = not significant

Suppl. Table 3. Psychological assessment over time - sub-group analysis of wwMS with available pre-pregnancy timepoint (not-imputed data)

Score	Parameters	Pre	Tri 1	Tri 2	Tri 3	2 wpp	3 mpp
modIDS	questionnaires	37	35	37	35	34	31
	mean (SD)	10.5 (8.9)	9.4 (8.3)	8.2 (5.9)	9.1 (6.4)	9.6 (10.5)	8.4 (8.1)
	median	8.0	7.0	8.0	8.0	5.5	8.0
	range	0-38	0-30	0-20	0-23	0-50	0-39
PSS-14	questionnaires	40	35	38	37	39	37
	mean (SD)	21.9 (5.6)	20.9 (6.4)	21.1 (6.3)	20.9 (6.3)	21.1 (7.5)	20.0 (6.3)
	median	22.0	22.0	21.5	21.0	22.0	20.0
	range	10-32	9-34	11-40	11-34	6-41	8-33
MFIS	questionnaires	40	39	39	39	38	38
	mean (SD)	24.1 (20.3)	23.6 (17.5)	21.8 (14.7)	23.6 (16.9)	23.3 (21.4)	22.0 (18.6)
	median	16.0	22.0	25.0	27.0	20.5	17.5
	range	0-62	0-58	0-57	0-61	0-71	0-63

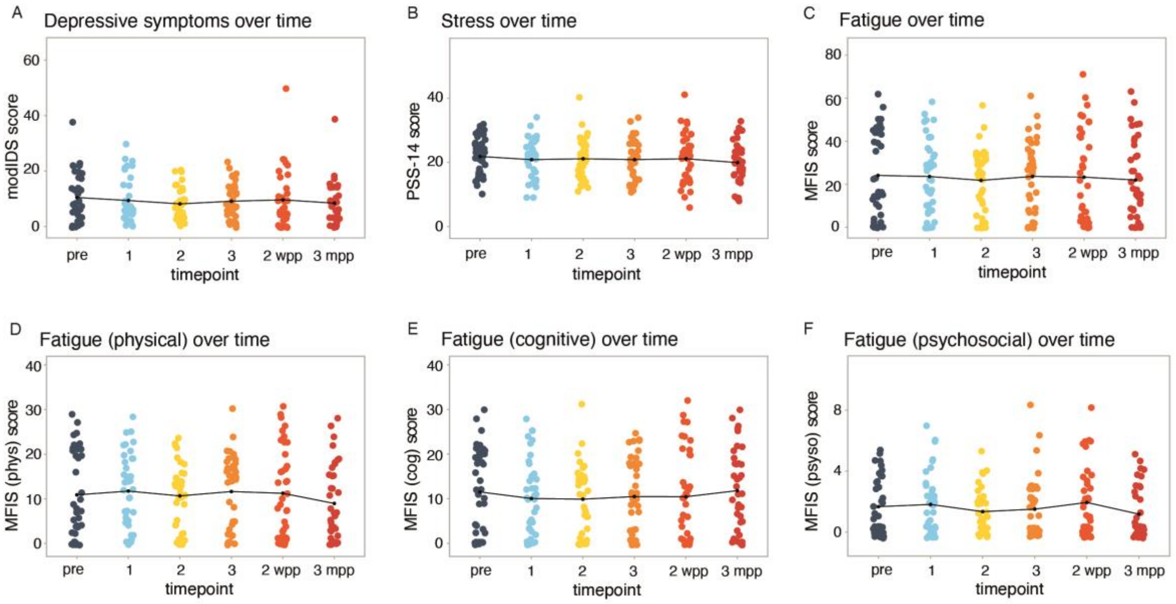

Suppl. Figure 2. Dynamics of depression, stress and fatigue over the course of pregnancy - sub-group analysis of wwMS with available pre-pregnancy timepoint (non-imputed data). Psychometric scores were assessed pre pregnancy (pre) and at tri 1 (1), tri 2 (2), tri 3 (3), 2 wpp and 3 mpp, with mean values per timepoint indicated by black curves: (A) modIDS, (B) PSS-14, (C) MFIS, MFIS (D) physical, (E) cognitive and (F) psychosocial subscores over time.

Suppl. Table 4. Analysis of psychometric score dynamics with LMM - sub-group analysis of wwMS with available pre-pregnancy timepoint (not-imputed data)

Timepoint	Estimate	Std. Error	df	t value	Pr(>|t|) adj	Significance level
**modIDS**						
**Intercept (Pre)**	10.46	1.34	72.43	7.79	(2.16^{-10})	(***)
**Timepoint 2 (Tri 1)**	-0.63	1.19	167.17	-0.53	0.594	ns
**Timepoint 3 (Tri 2)**	-2.30	1.17	166.97	-1.97	0.102	ns
**Timepoint 4 (Tri 3)**	-1.63	1.19	167.26	-1.37	0.258	ns
**Timepoint 5 (2 wpp)**	-1.23	1.20	167.41	-1.026	0.367	ns
**Timepoint 6 (3 mpp)**	-2.69	1.23	167.73	-2.185	0.090	ns
**PSS-14**						
**Intercept (Pre)**	21.85	1.01	100.67	21.57	$(<2^{-16})$	(***)
**Timepoint 2 (Tri 1)**	-1.32	1.05	182.05	-1.26	0.381	ns
**Timepoint 3 (Tri 2)**	-0.83	1.02	181.58	-0.81	0.471	ns
**Timepoint 4 (Tri 3)**	-1.18	1.03	181.73	-1.15	0.381	ns
**Timepoint 5 (2 wpp)**	-0.73	1.02	181.36	-0.72	0.471	ns
**Timepoint 6 (3 mpp)**	-1.98	1.03	181.67	-1.92	0.168	ns
**MFIS**						
**Intercept (Pre)**	24.07	2.90	60.50	8.30	(8.55^{-11})	(***)
**Timepoint 2 (Tri 1)**	-0.78	2.04	188.07	-0.38	0.841	ns
**Timepoint 3 (Tri 2)**	-2.30	2.04	188.07	-1.12	0.526	ns
**Timepoint 4 (Tri 3)**	-0.85	2.04	188.10	-0.42	0.841	ns
**Timepoint 5 (2 wpp)**	-0.41	2.06	188.13	-0.20	0.841	ns
**Timepoint 6 (3 mpp)**	-3.10	2.06	188.16	-1.51	0.402	ns

Abbreviations: df = degrees of freedom, Pr(>|t|) adj = *p* value corresponding to t-statistic for each fixed effect (FDR adjustment with Benjamini-Hochberg correction), Std. error = standard error. Significance levels: * = $p < 0.05$, ** = $p < 0.01$, *** = $p < 0.001$, ns = not significant
